# Phenolic compounds disrupt spike-mediated receptor-binding and entry of SARS-CoV-2 pseudo-virions

**DOI:** 10.1371/journal.pone.0253489

**Published:** 2021-06-17

**Authors:** Anna Goc, Waldemar Sumera, Matthias Rath, Aleksandra Niedzwiecki

**Affiliations:** Department of Infectious Diseases, Dr. Rath Research Institute, San Jose, California, United States of America; University of Melbourne, AUSTRALIA

## Abstract

In the pursuit of suitable and effective solutions to SARS-CoV-2 infection, we investigated the efficacy of several phenolic compounds in controlling key cellular mechanisms involved in its infectivity. The way the SARS-CoV-2 virus infects the cell is a complex process and comprises four main stages: attachment to the cognate receptor, cellular entry, replication and cellular egress. Since, this is a multi-part process, it creates many opportunities to develop effective interventions. Targeting binding of the virus to the host receptor in order to prevent its entry has been of particular interest. Here, we provide experimental evidence that, among 56 tested polyphenols, including plant extracts, brazilin, theaflavin-3,3’-digallate, and curcumin displayed the highest binding with the receptor-binding domain of spike protein, inhibiting viral attachment to the human angiotensin-converting enzyme 2 receptor, and thus cellular entry of pseudo-typed SARS-CoV-2 virions. Both, theaflavin-3,3’-digallate at 25 μg/ml and curcumin above 10 μg/ml concentration, showed binding with the angiotensin-converting enzyme 2 receptor reducing at the same time its activity in both cell-free and cell-based assays. Our study also demonstrates that brazilin and theaflavin-3,3’-digallate, and to a still greater extent, curcumin, decrease the activity of transmembrane serine protease 2 both in cell-free and cell-based assays. Similar pattern was observed with cathepsin L, although only theaflavin-3,3’-digallate showed a modest diminution of cathepsin L expression at protein level. Finally, each of these three compounds moderately increased endosomal/lysosomal pH. In conclusion, this study demonstrates pleiotropic anti-SARS-CoV-2 efficacy of specific polyphenols and their prospects for further scientific and clinical investigations.

## Introduction

The SARS-CoV-2 strain, also known as the 2019 novel coronavirus (2019-nCoV), belongs to the genus *Betacoronavirus* of the *Coronaviridae* family, and has been identified as a cause of respiratory infection characteristic of COVID-19 disease, declared a pandemic by the World Health Organization (WHO) in 2020 [1]. According to the US National Institutes of Health (NIH), this strain is closely related to the SARS-CoV-1 (SARS-CoV) strain that was responsible for outbreaks in 2002–2004 in Asia [2–5]. With a genome size of ~ 30 kilobases, which encodes structural proteins such as spike (S) protein, envelope (E) protein, membrane (M) protein, and the nucleocapsid (N) protein, SARS-CoV-2 is a positive-sense, single-stranded RNA virus that invades human cells through binding of its distinct surface spike protein (S glycoprotein) to a specific receptor present on the membrane of cells [3–5]. This attachment mediates viral host-cell membrane fusion and endocytic entry [5, 6].

The spike protein is a transmembrane protein with an N-terminal domain (NTD) and a C-terminal domain (CTD). The N-terminal domain, or S1 subunit, contains receptor-binding domain (RBD), while the C-terminal, or S2 subunit, is characterized by two heptad-repeat (HR) regions, which, upon assembly, induce membrane fusion and viral entry to the host cell [5–9]. Zhou *et al*. and Hoffman *et al*., among others, demonstrated that SARS-CoV-2 binds to human angiotensin-converting enzyme 2 (hACE2), a monomeric transmembrane protein present on many types of human cells [10–13]. Since spike protein governs viral attachment and virus-cell membrane fusion, which subsequently determines the fate of viral replication and infectivity, it has therefore been of the interest as a therapeutic target [9].

It has further been shown that binding of spike protein to the ACE2 receptor, and viral entry, require priming of spike protein at the S1/S2 surface units and the S2 site by host cellular proteases. This two-phase enzymatic cleavage allows fusion of viral and cellular membranes, and is essential for viral pathogenesis, which is activated by a host membrane protease, furin [8, 14–17]. In addition, Liu *et al*. and Glowacka *et al*. demonstrated that the endosomal cysteine protease cathepsin L, and transmembrane serine protease 2 (TMPRSS2), play a key role in in the entry process [18, 19]. Cathepsin L, as a member of the papain-like lysosomal family of acidic cysteine proteinases, is an endopeptidase expressed in almost all human eukaryotic cells as a 43 kDa pro-enzyme, which is further converted to mature form, influenced by cell-cell contact and extracellular matrix (ECM) components such as heparan sulfate, a glycosaminoglycan [20, 21]. Interestingly, reports of increased cathepsin L activity in the lung epithelial lining fluid of emphysema patients have led to the suggestion that this enzyme may be a determinant in the progression of SARS-CoV-2 infection as well [22].

The other enzyme associated with SARS-CoV-2 infection, TMPRSS2, belongs, as already noted, to the serine protease family. It contains a type II transmembrane domain, a receptor class A domain, a scavenger receptor cysteine-rich domain and a protease domain [23]. Earlier reports indicated differences in TMPRSS2 expression in the lung cells across different populations. Gender is not a factor, according to several further studies, which have suggested that constitutive expression of TMPRSS2 in lung cells does not differ between males and females [24, 25]. However, a report by Bertram *et al* suggested that TMPRSS2 is less expressed in Type II alveolar cells and alveolar macrophages than in bronchial epithelial cells [26, 27]. This study also demonstrated no expression of TMPRSS2 protein in Type I alveolar cells of the respiratory surface. These findings are of particular interest considering the putative role of TMPRSS2 in SARS-CoV-2 infection [11].

Polyphenols are one of the most important and certainly the largest among the groups of phytochemicals present in the plant kingdom, with a broad spectrum of properties affecting physiological and biochemical processes [28–30]. This vast group of bioactive compounds is divided into six major classes: hydroxybenzoic acids, hydroxycinnamic acids, flavonoids, stilbenes, and lignans. Flavonoids are further divided also into subgroups, which include flavonols, flavones, isoflavones, flavanones, anthocyanidins, and flavanols. Many polyphenols have shown therapeutic efficacy in various aspects of human health [31]. It is also a well-known fact that their adequate intake may help to modulate immune responses and resistance to infection. The efficacy of polyphenols as antiviral compounds has been frequently reported, and there is an enormous potential in exploring their antiviral properties, since they are commonly recognized as safe and effective in substituting for, or in serving as an adjunct treatment to, conventional therapies [32–50]. Although, there is already substantial information about polyphenols’ activity against SARS-CoV-2, most of these results are derived from computational modeling and computational predictions, and their capability as anti-SARS-CoV-2 agents still needs to be scientifically and clinically evaluated.

Here, we present experimental results showing a potential of representative polyphenols to inhibit the binding and entry of SARS-CoV-2 virions. Using standard and recently developed methodology, we report that, among 56 tested phenolic compounds, including plant extracts, brazilin, TF-3, and curcumin have the highest binding affinity to the viral RBD of SARS-CoV-2 spike protein. Moreover, concurrent experiment with SARS-CoV-2 pseudo-viral particles revealed that these three polyphenols have the pronounced inhibitory effect on viral binding and cellular entry. We also discovered that TF-3 and curcumin inhibit the activity of TMPRSS2 and cathepsin L proteases that facilitate the binding and endosomal egress of SARS-CoV-2, and modestly increase lysosomal pH, as does brazilin. In conclusion, this study documents anti-SARS-CoV-2 activity of these three polyphenols, providing a scientific basis for their further investigations in *in vivo* and clinical studies.

## Materials and methods

### Cell lines and pseudo-viruses

Human alveolar epithelial cell line A549 was obtained from ATCC (American Type Culture Collection) (Manassas, VA). Human alveolar epithelial cell line A549, stably overexpressing hACE2 receptor (hACE2/A549), and eGFP-luciferase-SARS-CoV-2 spike glycoprotein pseudo-typed particles were obtained from GenScript (Piscataway, NJ). Cell lines were cultured in Dulbecco’s Modified Eagle’s Medium (DMEM) supplemented with 10% fetal bovine serum (FBS), 100 U/ml penicillin, and 100 μg/ml streptomycin. Pseudo-typed ΔG-luciferase (G*ΔG-luciferase) rVSV was purchased from Kerafast (Boston, MA). Bald pseudo-virus particles with eGFP and luciferase (eGFP-luciferase-SARS-CoV-2 pseudo-typed particles) were purchased from BPS Bioscience (San Diego, CA). Lentiviral particles encoding human TMPRSS2 were from Addgene (Watertown, MA).

### Test compounds, antibodies, recombinant proteins and inhibitors

Curcumin, tea extract standardized to 85% theaflavins, theaflavin-3,3’-digallate, gallic acid, tannic acid, *Andrographis paniculata* extract, andrographolide, licorice extract, glycyrrhizic acid, broccoli extract, L-sulforaphane, usnic acid, malic acid, D-limonene, and ammonia chloride with purity between 95–99%, according to the manufacturer, were purchased from Sigma (St. Louis, MO). All other polyphenols and camostat mesylate, with purity between 95–99% according to the manufacturer, were obtained from Cayman Chemical Company (Ann Arbor, MI). For screening study, test compounds were prepared as 10 mg/ml (25% DMSO) working stock solution and for the rest of experiments as 1.0 mg/ml (1% DMSO) and 10 mg/ml (10% DMSO). All antibodies were from Santa Cruz Biotechnology (Santa Cruz, CA). TMPRSS2 recombinant protein was from Creative BioMart (Shirley, NY).

### Receptor binding and entry assays

#### SARS-CoV-2 RBD binding to hACE2

Binding reaction was performed using a SARS-CoV-2 Surrogate Virus Neutralization Test Kit that can detect either antibodies or inhibitors that block the interaction between the RBD-SARS-CoV-2 spike protein with the hACE2 receptor (GenScript, Piscataway, NJ). For screening, phenolic compounds or plant extracts (at 100 μg/ml concentration) were incubated with HRP-conjugated RBD-SARS-CoV-2 spike S1 domain for 30 min. at 37°C. Next, the samples that were incubated with RBD were transferred into a 96-well plate with immobilized hACE2 receptor and incubated for additional 15 min. at 37°C. Subsequently, the plates were washed four times with washing buffer and developed with TMB substrate solution for up to 5 min., followed by the addition of stop buffer. Optical density was measured immediately at 450 nm with a plate reader (Molecular Devices, San Jose, CA). Positive and negative controls were provided by the manufacturer. Control was 0.25% DMSO. Results are expressed as a percentage of polyphenol-free control (mean +/- SD, n = 6).

#### SARS-CoV-2 pseudo-virus binding to hACE2

Binding reaction was performed using a GenScript-developed protocol with small applied adjustments. Briefly, eGFP-luciferase-SARS-CoV-2 spike S1 pseudo-virus was either pre-incubated at 37°C with selected polyphenols (i.e., brazilin, TF-3, and curcumin) at concentrations ranging from 0–25 μg/ml for: 1) 1h before adding into a plate with hACE2/A549 cells, 2) simultaneously added into the plate with hACE2/A549 cells, or 3) added into the plate with the hACE2/A549 cells 1h post-treatment. A parallel experiment was performed, in which eGFP-luciferase-CoV-2 spike protein enveloped pseudo-virus was spin-inoculated at 1,200 x g for 45 min. Samples were incubated for an additional 1h, 3h, and 48h, at 37°C. After the incubation period, the plates were washed three times with washing buffer (provided by the manufacturer), and measured either HRP signal or luciferase activity using a Luciferase Glo Kit (Promega, Madison, WI). In 1h and 3h experiments, positive and negative controls were the same as those used in SARS-CoV-2 RBD binding to hACE2 assay, and were provided by the manufacturer. In 48h experiments, the positive control was bald eGFP-luciferase-SARS-CoV-2 pseudo-typed particles, and the negative control was ΔG-luciferase rVSV pseudo-typed particles. Control was 0.025% DMSO. Results are expressed as a percentage of polyphenol-free control (mean +/- SD, n = 6).

#### SARS-CoV-2 spike-protein-expressing cells binding to soluble hACE2

To transduce cells with eGFP-luciferase-SARS-CoV-2 spike S1 lentivirus vector (GenScript, Piscataway, NJ), A549 cells, seeded into a 6-well plate in the presence of complete growth medium, were treated with 8 μl/ml polybrene (Sigma, St. Louis, MO) for 30 min., followed by the addition of eGFP-luciferase-SARS-CoV-2 spike S1 lentivirus at MOI = 40 [51], and spin-inoculation at 1,000 x g. for 1.5h. After 24h at 37°C incubation, cells were fed with fresh complete growth medium. After 48h post-inoculation, cells were detached with 1 mM EDTA, washed twice with 1 x PBS (phosphate-buffered saline) supplemented with 3% FBS, and treated with indicated concentrations of polyphenols for 1h, followed by incubation with 5 μg/ml of soluble hACE2 (Sigma, St. Louis, MO) for 1h on ice. After washing three times with 3% FBS in 1 x PBS, cells were transferred into plates with human monoclonal anti-ACE2 antibody at 10 μg/ml (Cayman Chemical Company, Ann Arbor, MI). After 1h incubation, wells were washed three times with 3% FBS in 1 x PBS, and fluorescence was measured at Ex/Em = 488/535 nm wavelength with a plate reader (Tecan Group Ltd, Switzerland). Positive and negative controls were the same as those used in SARS-CoV-2 RBD binding to hACE2 assay, and were provided by the manufacturer. Control was 0.025% DMSO. Results are expressed as a percentage of polyphenol-free control (mean +/- SD, n = 6).

### Cell-cell fusion assay

Cell-cell fusion assay was performed according to Ou *et al*. [13]. Briefly, A549 cells transduced with eGFP-luciferase-SARS-CoV-2 spike S1 lentivirus vector (GenScript, Piscataway, NJ) were detached with 1 mM EDTA, treated with indicated concentrations of selected polyphenols, for 1h at 37°C, and overlaid on 80–95% confluent human A549 lung epithelial cells overexpressing hACE2. After 4h incubation at 37°C, images of syncytia were captured with a Zeiss AxioObserver A1 fluorescence microscope (Carl Zeiss Meditec, Dublin, CA). The positive control was 20 μg/ml anti-ACE2 antibody. Control was 0.025% DMSO. Results are expressed as a percentage of polyphenol-free control (mean +/- SD, n = 3).

### TMPRSS2 activity assay

Cellular TMPRSS2 activity assay was performed according to a previously published report [52]. Briefly, hTMPRSS2/A549 cells were seeded in 48-well plates. 48h or 3h prior to the protease activity measurements, the cells were treated with selected polyphenols at 5.0–25 μg/ml concentrations. Next, cells were washed with DMEM without phenol red, and the protease activity was assessed by incubation of cells with the 200 μM fluorogenic substrate Mes-D-Arg-Pro-Arg-AMC in 50 mM PBS (pH = 7.4) for 30 min. at 37°C (Fisher Scientific, Pittsburgh, PA). Hydrolysis of the peptide was monitored by the measurement of fluorescence intensity, using a spectrofluorometer at Ex/Em = 360/440 nm wavelength (Tecan Group Ltd, Switzerland). The positive control was 50 μM camostat mesylate. Control was 0.025% DMSO. Results are expressed as a percentage of polyphenol-free control (mean +/- SD, n = 6).

Direct TMPRSS2 activity assay with recombinant enzyme was performed according to a previously published report [53]. To determine the inhibitory effect of selected polyphenols on the activity of isolated TMPRSS2 protein, 1 μM fluorogenic peptide Boc-Gln-Ala-Arg-AMC was added to the selected polyphenols diluted at 5.0–25 μg/ml concentrations. To this reaction 10 μM of TMPRSS2 enzyme in assay buffer (50 mM Tris pH = 8, 150 mM NaCl) was added. Following 1h incubation at RT, detection of fluorescent signal was performed using a spectrofluorometer at Ex/Em = 360/440 nm wavelength (Tecan Group Ltd, Switzerland). The positive control was 100 μM camostat mesylate. Control was 0.025% DMSO. Results are expressed as a percentage of polyphenol-free control (mean +/- SD, n = 6).

### Cathepsin L activity assays

Cellular cathepsin L activity assays were performed utilizing a Cathepsin L Activity Assay Kit (Abcam, Cambridge, MA) according to the manufacturer’s protocol. Briefly, A549 cells were seeded in 6-well plates and allowed to adhere for 24h or until reaching 90–95% of confluence. Next, the cells were treated with indicated concentrations of selected polyphenols for an additional 24h, washed with cold 1 x PBS, and lysed using 100 μl of chilled CL buffer on ice for 5 min. The samples were then centrifuged for 2 min. at 4°C to remove any insoluble material. Supernatants were collected and transferred to clean tubes that were kept on ice. Enzymatic reaction was set up by mixing treated sample wells containing 50 μl sample, 50 μl untreated sample (control), 50 μl background control, a positive control containing 5 μl reconstituted positive control in 45 μl CL buffer, and a negative control containing 5 μl reconstituted positive control in 45 μl CL buffer and 2 μl CL inhibitor. Next, 50 μl CL buffer and 1 μl 1 mM DTT were added to each well. Finally, 2 μl 10 mM CL substrate Ac-FR-AFC (200 μM final concentration) was added to each well, except background control samples. The plates were incubated at 37°C for 1h and fluorescence signal was measure at Ex/Em = 400/505 nm wavelength with a spectrofluorometer (Tecan Group Ltd, Switzerland). Control was 0.025% DMSO. Results are expressed as a percentage of polyphenol-free control (mean +/- SD, n = 6).

The inhibitory effect of selected polyphenols on the activity of isolated cathepsin L enzyme was evaluated using a Cathepsin L Activity Screening Assay Kit (Abcam, Cambridge, MA) according to the manufacturer’s protocol. Selected polyphenols at 0.25–2.5 μg/ml concentrations were added to cathepsin L (0.2 mU/μl) and the reaction mix was incubated for 15 min. at RT. The positive control was the sample containing only cathepsin L, and the negative control was a sample containing cathepsin L and cathepsin L inhibitor (FF-FMK) (10 μM). Cathepsin L substrate (Ac-FR-AFC) (10 mM) was added to each well, and the plate was incubated for 30 min. at 37°C. The fluorescence was measured at Ex/Em = 405/505 nm wavelength using a spectrofluorometer (Tecan Group Ltd, Switzerland). Control was 0.025% DMSO. Results are expressed as a percentage of polyphenol-free control (mean +/- SD, n = 6).

### ACE2 activity assays

To determine the inhibitory effect of selected polyphenols on the activity of cellular hACE2 protein, hACE2/A549 cells were seeded in 48-well plates and allowed to adhere for 24h or until reaching 99–100% of confluence. The cells were then treated with indicated concentrations of selected polyphenols for an additional 24h, before being washed with cold 1 x PBS, and enzymatic reaction was initiated by adding 200 μM fluorogenic substrate Mca-Y-V-A-D-A-P-K(Dnp)-OH. Finally, the plates were incubated at 37°C for 1h, and the fluorescence signal was measured at Ex/Em = 320/405 nm wavelength with a spectrofluorometer (Tecan Group Ltd, Switzerland). Control was 0.025% DMSO. Results are expressed as a percentage of polyphenol-free control (mean +/- SD, n = 6).

To determine the inhibitory effect of selected polyphenols on the activity of recombinant hACE2 protein, an ACE2 Activity Screening Assay Kit (BPS Bioscience, San Diego, CA) was used according to the manufacturer’s protocol. Briefly, to ACE2 enzyme (0.2 mU/μl) the selected polyphenols at 5.0–25 μg/ml concentrations were added and the reaction mix was incubated for 15 min. at RT. The positive control was a sample containing only ACE2 enzyme, and the negative control was a sample containing ACE2 enzyme and 10% DMSO. ACE2 fluorogenic substrate (10 μM) was added to each well, and the plate was incubated for 1h at RT. The fluorescence was measured at Ex/Em = 535/595 nm wavelength using a spectrofluorometer (Tecan Group Ltd, Switzerland). Control was 0.025% DMSO. Results are expressed as a percentage of polyphenol-free control (mean +/- SD, n = 6).

### ACE2 binding assay

To determine the inhibitory effect of selected polyphenols on binding to the ACE2 receptor, an ACE2 Inhibitor Screening Assay Kit (BPS Bioscience, San Diego, CA) was used according to the manufacturer’s protocol. Briefly, to ACE2 receptor immobilized on the plate (1.0 μg/ml), selected polyphenols at 5.0–25 μg/ml concentrations were added and the reaction mix was incubated for 1h at RT. The positive control contained 20 μg/ml anti-ACE2 antibody in the sample, and the negative control was an addition-free sample. Next, the plate was washed three times with washing buffer, and SARS-CoV-2 spike protein at 1.0 μg/ml was applied for 1h at RT, followed by washing three times, blocking with blocking buffer, and incubation with HRP-conjugated secondary antibody for an additional 1h at RT. The plates were again washed three times with washing buffer, and chemiluminescence was measured using ECL substrate A and ECL substrate B mixed 1:1, using a micro-plate reader (Tecan Group Ltd, Switzerland). Control was 0.025% DMSO. Results are expressed as a percentage of polyphenol-free control (mean +/- SD, n = 6).

### Endosomal/lysosomal pH assay

Endosomal pH was assessed according to a previously reported protocol [54]. Briefly, A549 cells were seeded in 8-well chambers (MatTek, Ashland, MA), and, at 95–100% confluence, were treated with the indicated polyphenols at 5.0 and 25 μg/ml concentrations, followed by 3h incubation at 37°C in a 5% CO_2_. Acridine orange (Thermo Fisher Scientific, Waltham, MA) was added directly to each dish to reach a final concentration of 6.6 μg/ml. The cells were additionally incubated at 37°C with 5% CO_2_ for 20 min. and washed three times with 1 x PBS. Live Cell Imaging Solution (LCIS) (Thermo Fisher Scientific, Waltham, MA) was added to the wells, and images were taken using a Zeiss Axio Observer A1 fluorescence microscope with a 40x magnification. Control was 0.025% DMSO, whereas the positive control was 20 mM ammonia chloride. Results are expressed as a percentage of polyphenol-free control (mean +/- SD, n = 3).

A concurrent experiment was performed using pHrodo^™^ Green AM Intracellular pH Indicator (Thermo Fisher Scientific, Waltham, MA) according to the manufacturer’s protocol. Briefly, A549 cells were seeded at 95–100% confluence, treated with the indicated polyphenols at 5 and 25 μg/ml concentrations, and incubated for 24h at 37°C with 5% CO_2_. Next, 10 μl of the pHrodo^™^ Green AM dye was added to 100 μl of PowerLoad^™^ to facilitates uniform cellular loading of AM esters, and the whole dye solution was transferred into 10 ml of LCIS. The growth medium from cells was removed, cells were washed once with LCIS, and replaced with the pHrodo^™^ Green AM staining solution. The plate was incubated for 30 min. at 37°C, washed again with LCIS, and fluorescence was measured at Ex/Em = 509/533 nm wavelength using a spectrofluorometer (Tecan Group Ltd, Switzerland). pH identification was performed based on standard curve, using an Intracellular pH Calibration Buffer Kit according to the manufacturer’s protocol (Thermo Fisher Scientific, Waltham, MA). Briefly, after performing cellular experiment with pHrodo^™^ Green AM, cells were washed twice with LCIS, the LCIS was replaced with cellular pH calibration buffer at pH = 4.5, supplemented with 10 μM of valinomycin and 10 μM of nigericin, and the cells were incubated at 37°C for 5 min. Next, the fluorescence was measured Ex/Em = 509/533 nm wavelength. These steps were repeated with the three additional cellular pH calibration buffers at pH = 5.5, 6.5 and 7.5, respectively, to obtain altogether four data points that were plotted to get the pH standard curve. Control was 0.025% DMSO, whereas the positive control was 20 mM ammonia chloride. The experiment was repeated three times, each one in triplicates.

### Viability assay

MTT assay was used to assess cell viability. Briefly, A549 cells were seeded into a 96-well plate at a cell density of 40,000 per well, and allowed to adhere for 24h, followed by treatment with different concentrations of selected polyphenols for up to 48h. Next, complete growth medium was replaced with a fresh one substituted with 5 mg/ml MTT, followed by incubation for 3h at 37°C. After removing the culture medium, 100 μl of methanol was added and the absorbance was measured at 570 nm using a spectrophotometer (Molecular Devices, San Jose, CA). Control was 0.025% DMSO. Results are expressed as a percentage of polyphenol-free control (mean +/- SD, n = 10).

### Western blot analysis

A549 cells were treated with indicated concentrations of selected polyphenols and lysed using RIPA lysis buffer (Sigma, St. Louis, MO) supplemented with 1X Complete protease inhibitors (Roche Applied Science, Indianapolis, IN). The protein concentration was measured by the Dc protein assay (Bio-Rad, Hercules, CA). Proteins (50 μg/well) were separated on 8–16% gradient SDS-PAGE gels and transferred to a PVDF membrane. Specific proteins were detected with commercially available human anti-cathepsin L, anti-TMPRSS2, and anti-ACE2 monoclonal antibodies, all at 1:200 dilution, and anti-β-actin antibody as a loading control at 1:1000 dilution. Images were captured with Azure^™^ cSeries digital imaging system (Azure Biosystems, Dublin, CA) with auto-exposure settings. Densitometry was performed with NIH ImageJ software.

### Statistical analysis

Data for all experiments are presented as an average value and standard deviation from at least three independent experiments. Comparison between different samples was done by a two-tailed T-test using the Microsoft Office Excel program. Differences between samples were considered significant at p values lesser than 0.05.

## Results

### Efficacy of phenolic compounds and plant extracts in preventing binding of RBD sequence of SARS-CoV-2 with hACE2 receptor

**Fig 1:**
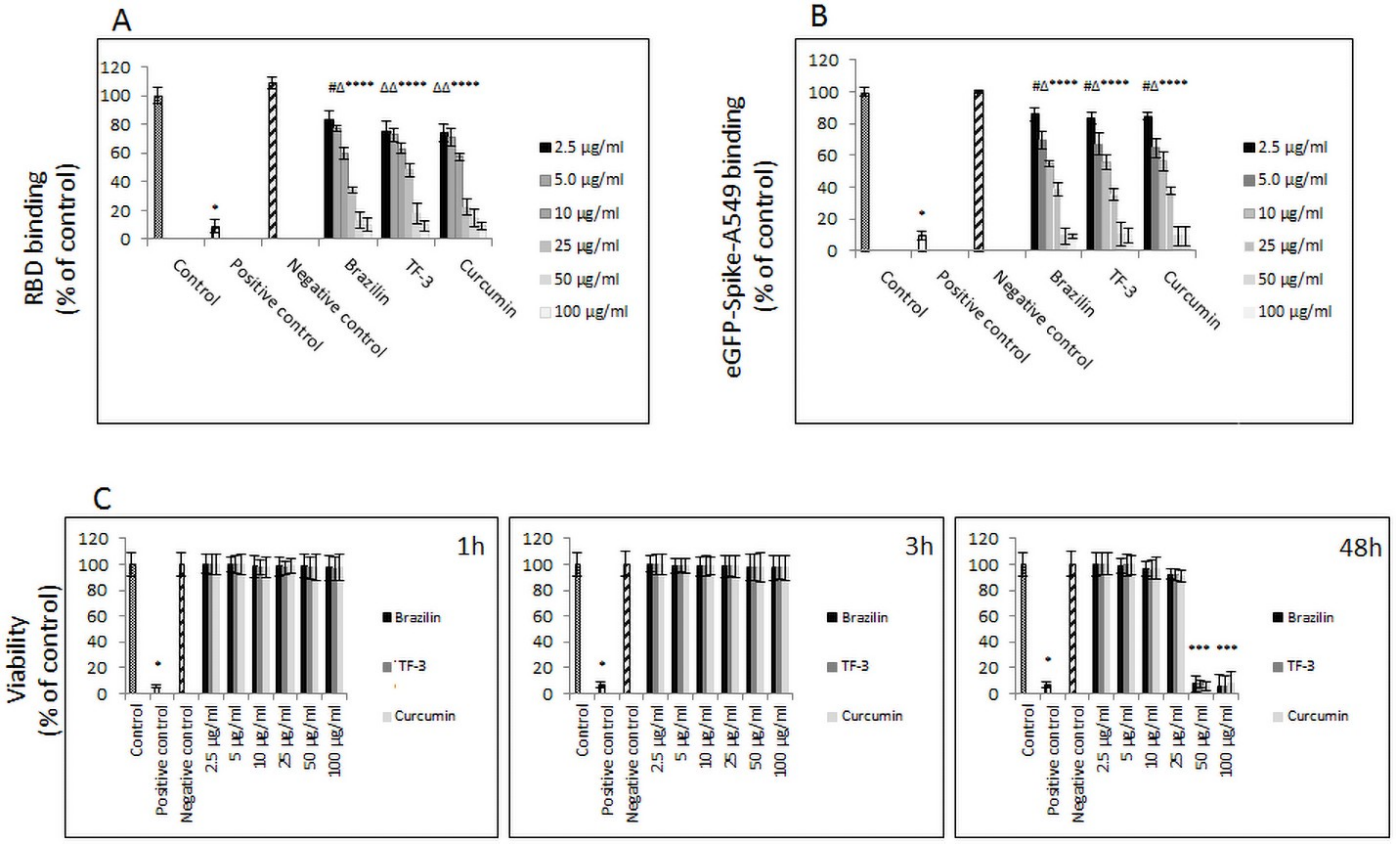
Binding of RBD-spike protein of SARS-CoV-2 to human ACE2 receptor. (A) Dose-dependent binding of RBD-SARS-CoV-2 to immobilized hACE2 receptor. Control– 0.025% DMSO, positive and negative controls were provided by the manufacturer; data are presented as % of control ± SD. (B) Dose-dependent binding of A546 cells expressing SARS-CoV-2 eGFP-spike protein, in the presence of indicated polyphenols at different concentrations, to soluble hACE2 receptor. Control– 0.25% DMSO; positive and negative controls were provided by the manufacturer; data are presented as % of control ± SD. (C) Viability of A549 cells, positive control—100% dead cells, negative control—addition-free sample; TF-3—theaflavin-3,3’-digallate; # p ≤ 0.05, Δ p ≤ 0.01, * p ≤ 0.001.

We investigated the ability of several classes of polyphenols to inhibit the binding of the RBD sequence of the SARS-CoV-2 spike protein to the hACE2 receptor, taking a two-stage approach. In the first approach we screened the capacity of 56 polyphenols and plant extracts, to inhibit binding of HRP-conjugated RBD-SARS-CoV-2 spike protein to the immobilized hACE2 receptor. As presented in Tables 1 and 2, three polyphenols: brazilin, TF-3, and curcumin showed the highest inhibitory effect at 100 μg/ml concentration. Moreover, the inhibitory effect of these most effective polyphenols, i.e., brazilin, TF-3, and curcumin, was dose dependent, ranging from 20% to 100% at 2.5–100 μg/ml, respectively (Fig 1A).

**Table 1:** Binding of various classes of phenolic compounds with RBD of SARS-CoV-2.

Tested polyphenols and alkaloids (0.1 mg/ml)	Binding with RBD (% of control±SD)
**Phenolic acids**	
Gallic acid	18.3±4.5
Tannic acid	79.4±2.3
Curcumin	**100±0.2**
Chlorogenic acid	25.5±2.5
Rosmarinic acid	22.5±3.8
**Flavonoids**	
Fisetin	22.4±1.9
Quercetin	22.4±6.5
Morin	30.5±5.8
Myricetin	45.5±5.4
Kaemferol	15.6±2.9
Rutin	20.6±6.3
Luteolin	10.4±4.7
Baicalein	22.5±5.1
Baicalin	10.3±2.9
Scutellarin	8.1±3.7
Naringin	23.6±6.4
Naringenin	20±5.1
Hesperidin	90.3±3.8
Hesperetin	42.5±4.6
Apigenin	17.1±4.1
Genistein	22.1±2.8
Phloroglucinol	69.5±3.6
Schizandrin	22.4±.3.3
Urolithin A	31.1±4.6
Punicalagin	32.3±5.9
Brazilin	**100±0.1**
Hispidulin	20.1±6.0
Papaverine	1.6±0.2
Silymarin	30.0±2.6
Procyanidin B2	31.1±3.6
Procyanidin B3	32.3±3.7
**Stilbenes**	
Trans-resveratrol	22.3±2.9
Pterostillbene	23.1**±**2.8
**Alkaloids**	
Palmatine	40.4±6.1
Berberine	17.3±2.7
Cannabidiol	1.4±0.3
Castanospermine	8.2±2.3
Usnic acid	22.0±3.4
Malic acid	1.2±3.7
**Terpenes**	
D-limonene	27.2±6.4
Carnosic acid	27.1±5.1

**Table 2:** Binding of selected plant extracts and their major components with RBD of SARS-CoV-2.

Tested plant extracts and their main active compounds (0.1 mg/ml)	Binding with RBD (% of control±SD)
Tea extract (85% catechin standardized)	88.3±3.7
(+)-gallocatechin	69.5±2.8
(-)-catechin gallate	37.4±4.7
(-)-gallocatechin gallate	75.4±5.6
(-)-gallocatechin	73.5±6.7
(+)-epigallocatechin gallate	87.5±6.8
Tea extract (85% theaflavins standardized)	**100±0.3**
Theaflavin	27.3±1.4
Theaflavin-3,3’-digallate	**100±0.1**
Broccoli extract	28.6±2.6
L-sulforaphane	30.2±3.6
*Andrographis paniculata* extract	18.4±1.8
Andrographolide	22.1±2.5
Licorice extract	18.3±3.6
Glycyrrhizic acid	22.2±2.3

In the second approach, we incubated A549 cells expressing SARS-CoV-2 spike protein with these three selected polyphenols for 1h and then exposed them to the soluble hACE2 receptor. In this experiment, we also observed dose-dependent interference ranging from 15% to 100% at 2.5–100 μg/ml, respectively, which corresponded to previously obtained results (Fig 1B).

A cell viability test revealed that short-term incubation (i.e., up to 3h) with these polyphenols at concentrations up to 100 μg/ml showed no cytotoxicity. However, with incubation time extended to 48 hours at doses of 50 μg/ml and above, decreased cell viability was noticed (Fig 1C).

### Effects of brazilin, theaflavin-3,3’-digallate and curcumin on binding and cellular entry of SARS-CoV-2 pseudo-virions

In subsequent experiments, we tested whether observed inhibitory effects of brazilin, TF-3, and curcumin, on RBD binding to hACE2, will persist when using SARS-CoV-2 viral particles. In these tests we used pseudo-virions enveloped with SARS-CoV-2 spike protein, and applied three different patterns, as follows: 1) SARS-CoV-2 virions carrying the genes for GFP-luciferase and pseudo-typed with the spike protein were incubated with selected polyphenols for 1h before being added to hACE2/A549 cells, 2) SARS-CoV-2 virions carrying the genes for GFP-luciferase and pseudo-typed with the spike protein were added simultaneously to hACE2/A549 cells, and 3) SARS-CoV-2 virions carrying the genes for GFP-luciferase and pseudo-typed with the spike protein were added to hACE2/A549 cells, and 1h after polyphenols were applied to the hACE2/A549 cells. Binding efficacy for each application pattern was evaluated after either 1h or 3h of incubation with the hACE2/A549 cells. Also, we evaluated the efficacy of these polyphenols after 48h post-infection, with or without spin-inoculation.

**Fig 2:**
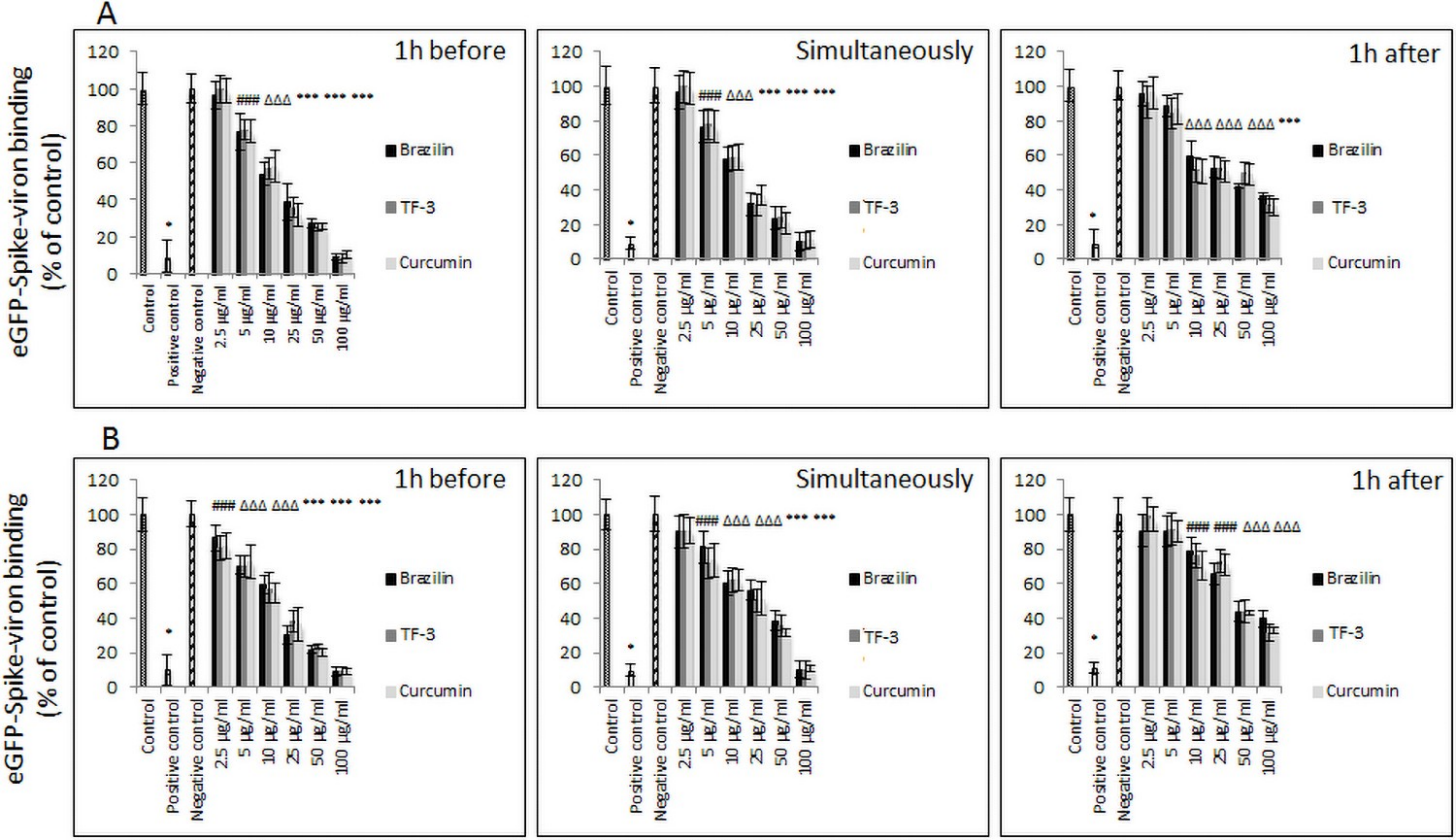
Binding of SARS-CoV-2 pseudo-virion to human ACE2 receptor. (A) Dose-dependent binding of SARS-CoV-2 spike protein-encapsulated pseudo-virions to A549 cells stably overexpressing human ACE2 receptor evaluated after 1h incubation. (B) Dose-dependent binding of SARS-CoV-2 spike protein-encapsulated pseudo-virions to A549 cells stably overexpressing hACE2 receptor evaluated after 3h incubation. Data are presented as % of control ± SD; control– 0.025% DMSO, positive and negative controls were provided by the manufacturer; TF-3 –theaflavin-3,3’-digallate # p ≤ 0.05, Δ p ≤ 0.01, * p ≤ 0.001.

Binding efficacy experiment revealed that brazilin, TF-3, and curcumin inhibit, in dose-dependent fashion, binding of SARS-CoV-2 spike protein pseudo-typed virions to hACE2/A549, regardless of exposure time and application pattern. This experiment also showed significant inhibition by these polyphenols, starting from 5.0 μg/ml, when 1h incubation was allowed (Fig 2A). With incubation extended to 3h, significant inhibition was observed from 2.5 μg/ml when SARS-CoV-2 virions were incubated with selected polyphenols for 1h before being added to hACE2/A549 cells. When SARS-CoV-2 virions were added simultaneously with selected polyphenols to hACE2/A549 cells, significant inhibition was noticed from 5.0 μg/ml, and from 10 μg/ml when selected polyphenols were applied in hACE2/A549 cells 1h after SARS-CoV-2 virions were applied (Fig 2B).

**Fig 3:**
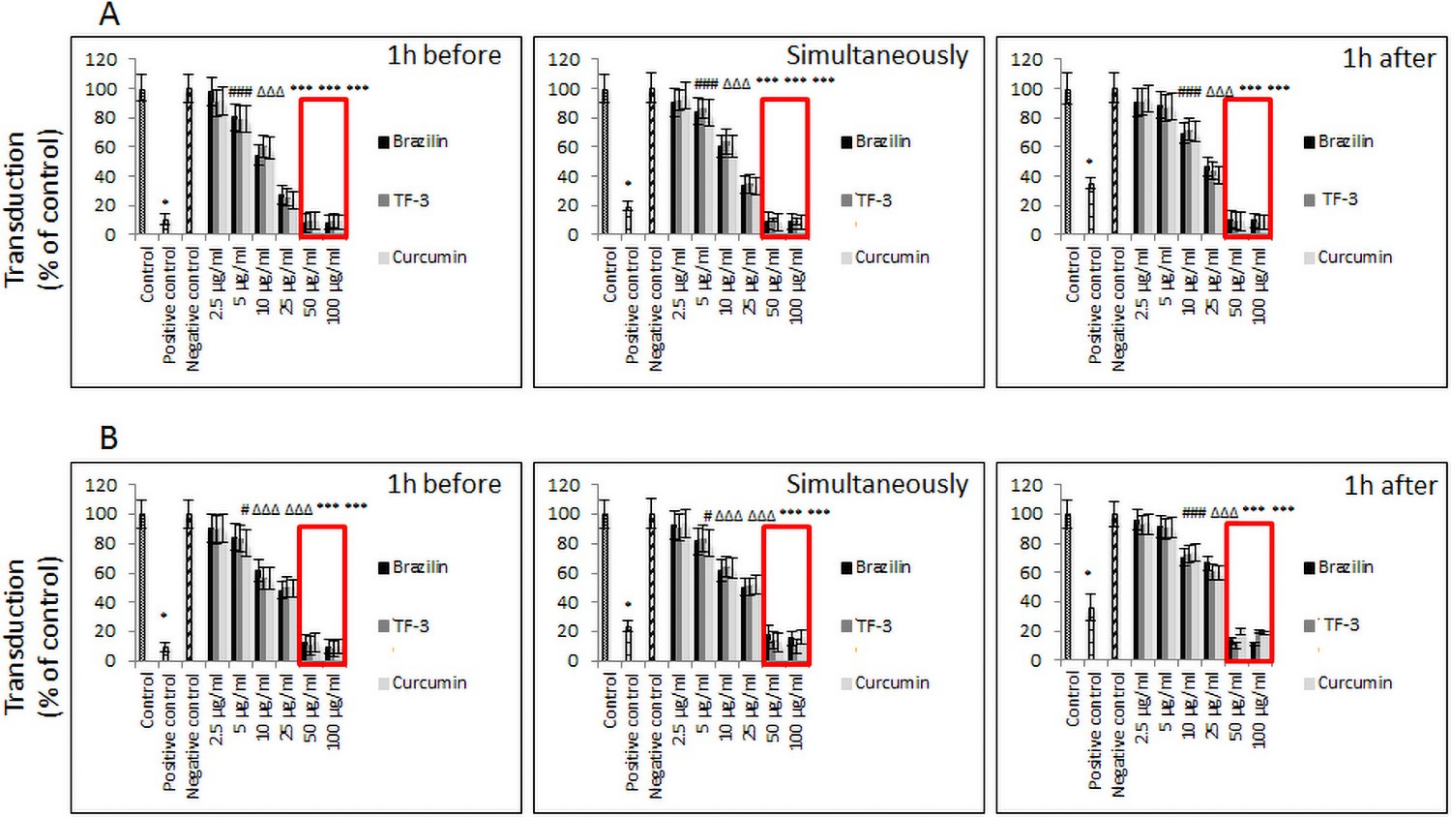
SARS-CoV-2 eGFP-luciferase-pseudo-virion cellular entry. (A) Attachment and entry of SARS-CoV-2 pseudo-virions with encapsulated eGFP-luciferase spike protein was evaluated without spinfection after 48h incubation. (B) Attachment and entry of SARS-CoV-2 pseudo-virions with encapsulated eGFP-luciferase spike protein was evaluated with spinfection after 48h incubation. Data are presented as % of control ± SD; TF-3 –theaflavin-3,3’-digallate # p ≤ 0.05, Δ p ≤ 0.01, * p ≤ 0.001. Control– 0.025% DMSO, positive control—bald SARS-CoV-2 eGFP-luciferase-pseudo-virions, negative control—ΔG-luciferase rVSV pseudo-typed particles; red fame—concentrations that showed 85–100% cytotoxicity.

The experiments, in which incubation was extended to 48h and whether or not spin-inoculation was applied, also revealed that brazilin, TF-3, and curcumin inhibit, in dose-dependent fashion, binding of SARS-CoV-2 spike protein pseudo-typed virions A549 to hACE2/A549 at non-toxic concentrations (i.e., 5.0–25 μg/ml). Inhibition ranged from 20% to 80% when spin-inoculation was not introduced, and from 20% to 40% when spin-inoculation was introduced (Fig 3). When spin-inoculation was not applied, significant inhibition was observed from 5.0 μg/ml concentration when SARS-CoV-2 spike pseudo-virions were either incubated with selected polyphenols 1h before hACE2/A549 cell exposure, or when SARS-CoV-2 spike pseudo-virions were added simultaneously with the tested polyphenols (Fig 3A). When the tested polyphenols were added 1h after SARS-CoV-2 pseudo-virions were applied, significant inhibition was noticed from 10 μg/ml concentration. When the viral binding to the hACE2/A549 cells was forced by application of spin-inoculation, significant inhibition was observed from 5.0 μg/ml when SARS-CoV-2 virions incubated with curcumin for 1h before being added to hACE2/A549 cells or when SARS-CoV-2 spike pseudo-virions were added simultaneously with curcumin. When SARS-CoV-2 virions were incubated with brazilin or TF-3, the inhibitory effect was observed from 10 μg/ml concentration. When test polyphenols were added 1h after SARS-CoV-2 virions were applied, significant inhibition was noticed from 10 μg/ml concentration (Fig 3B).

**Fig 4:**
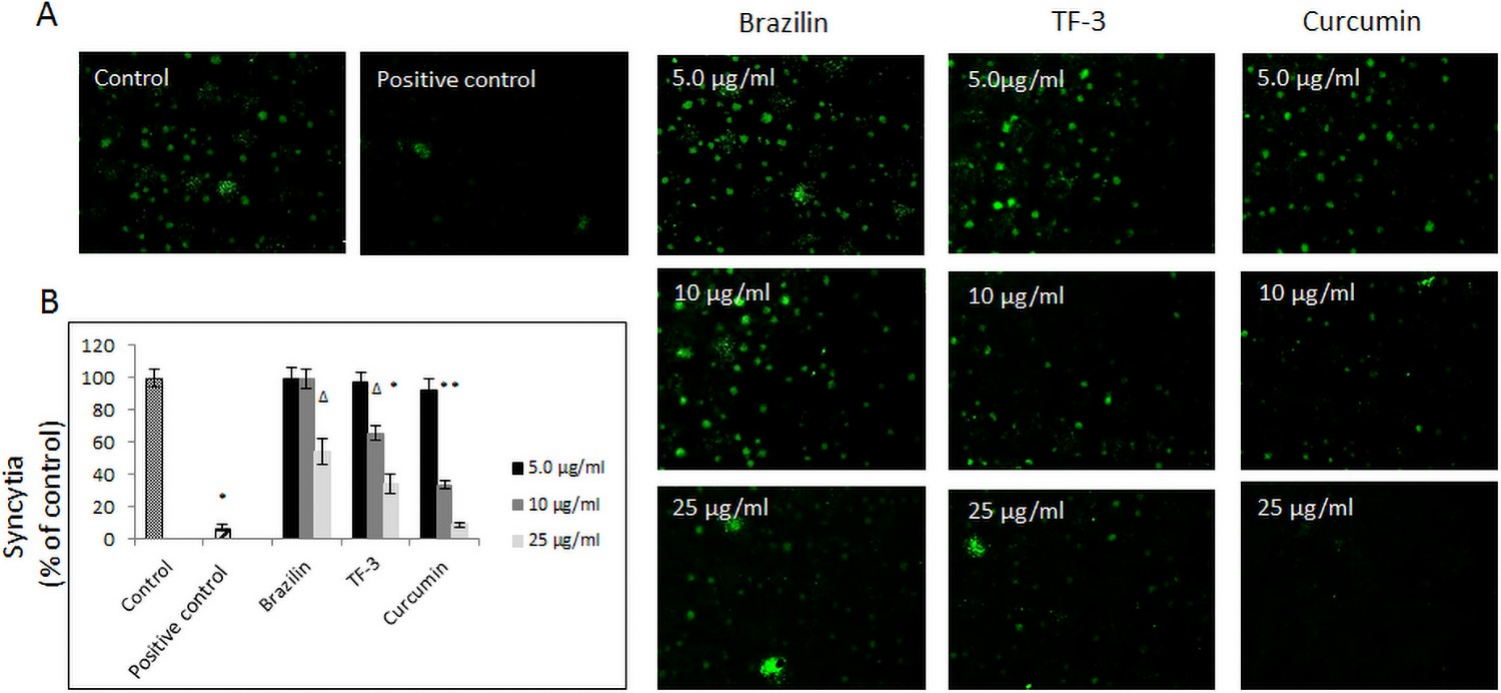
Effect of selected polyphenols on fusion to human ACE2 receptor overexpressing A549 cells. (A) Cell-cell fusion of A549 cells expressing eGFP spike protein with A549 cells stably expressing human ACE2 receptor. The scale bar indicates 250 μm. (B) Quantitative analysis of formed syncytia. Experiments were done in triplicate and repeated three times. Data are presented as % of control ± SD; TF-3 –theaflavin-3,3’-digallate Δ p ≤ 0.01, * p ≤ 0.001. Control– 0.025% DMSO, positive control– 20 μg/ml anti-ACE2 antibody.

Also, our further experiment, where A549 cells expressing SARS-CoV-2 spike protein pseudo-typed virions were pre-incubated with the tested polyphenols, and then layered for 4h on hCE2/A549, the cells showed a significantly decreased attachment. Incubation with brazilin at 25 μg/ml decreased the fusion by 40%, with TF-3 at 10–25 μg/ml by 40% to 70%, and with curcumin at the same concentrations, i.e., 10–25 μg/ml, by 70% to 95%. The results were consistent with previously obtained sets of data (Fig 4).

### Effect of brazilin, theaflavin-3,3’-digallate and curcumin on cellular proteases involved in entry and endosomal egress of SARS-CoV-2 pseudo-virions

**Fig 5:**
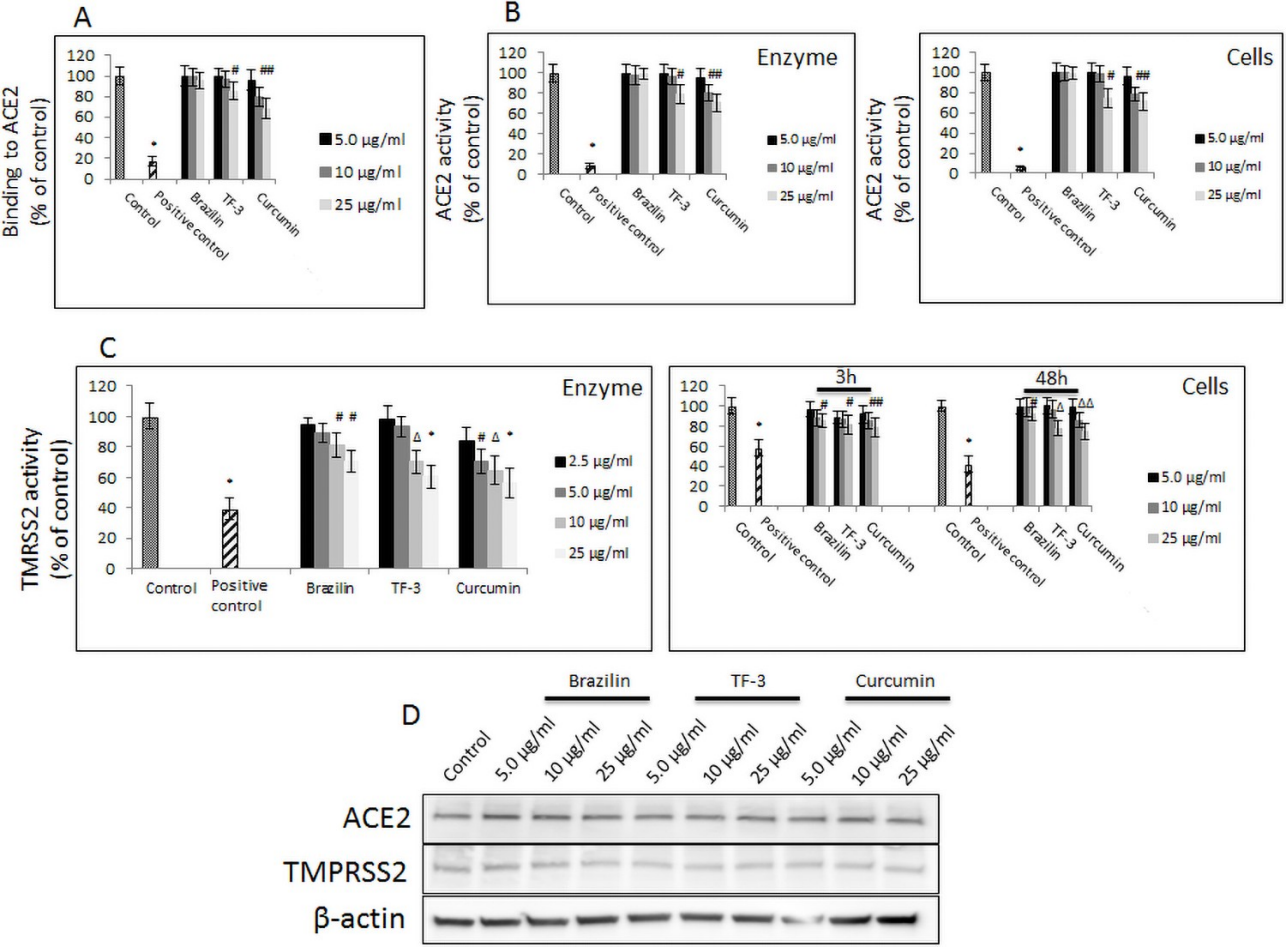
Effects of selected polyphenols on cellular membrane associated proteases. (A) Binding of indicated polyphenols at different concentrations to hACE2 receptor. Data are presented as % of control ± SD; control– 0.025% DMSO, positive control– 50% DMSO. (B) Activity of recombinant hACE2 upon treatment with indicated polyphenols at different concentrations. (left panel). Activity of cellular hACE2 upon treatment with indicated polyphenols at different concentrations. (right panel). Data are presented as % of control ± SD; * p ≤ 0.001. Control– 0.025% DMSO, positive control– 10% DMSO. (C) Activity of recombinant TMPTSS2 upon treatment with indicated polyphenols at different. (left panel). Activity of cellular TMPTSS2 upon treatment with indicated polyphenols at different concentrations (right panel). Data are presented as % of control ± SD; # p ≤ 0.05, Δ p ≤ 0.01, * p ≤ 0.001. Control– 0.025% DMSO, positive control– 50–100 μM camostat mesylate. (D) Western blot analysis of hACE2 and TMPRSS2 expression in A549 cells upon treatment with indicated polyphenols with different concentration for 48h period. Data are presented as % of control ± SD; control– 0.025% DMSO, TF-3 –theaflavin-3,3’-digallate.

The crucial step in the SARS-CoV-2 virions internalization involves the cognate ACE2 receptor. Therefore, we checked whether or not brazilin, TF-3, and curcumin affect binding to and activity of the ACE2 molecule itself. Our results showed that brazilin does not bind to ACE2 directly, in contrast to TF-3 and curcumin, which showed binding efficacy at 25 μg/ml and at 10–25 μg/ml, respectively. In addition, we observed minor 20%-30% inhibition of ACE2 activity in both cell-free and cell-based assays with TF-3 at 25 μg/ml and curcumin at 10–25 μg/ml, respectively, and no effects with brazilin (Fig 5A and 5B).

In order to gain deeper insight into the mechanism by which these three polyphenols suppress the SARS-CoV-2 virions cellular penetration, and knowing that the SARS-CoV-2 virions internalize via an endocytic pathway, but that, at the same time, host cellular proteases are involved, we checked the activity and cellular expression of TMPRSS2. As shown in Fig 5C, significant inhibition of recombinant hTMPRSS2 activity was observed, upon 3h treatment with brazilin and TF-3 at 10–25 μg/ml, ranging from 20–30% for brazilin, and from 30% to 40% for TF-3, whereas curcumin treatment decreased TMPRSS2 activity by about 40% to 50%. Activity of hTMPRSS2 overexpressed on A549 cells was also affected by these compounds upon 48h treatment that followed the pattern observed in short-term experiment (i.e., 3h treatment). Our results also showed that expression of of ACE2 and TMPRSS2 at protein level was not affected (Fig 5D).

**Fig 6:**
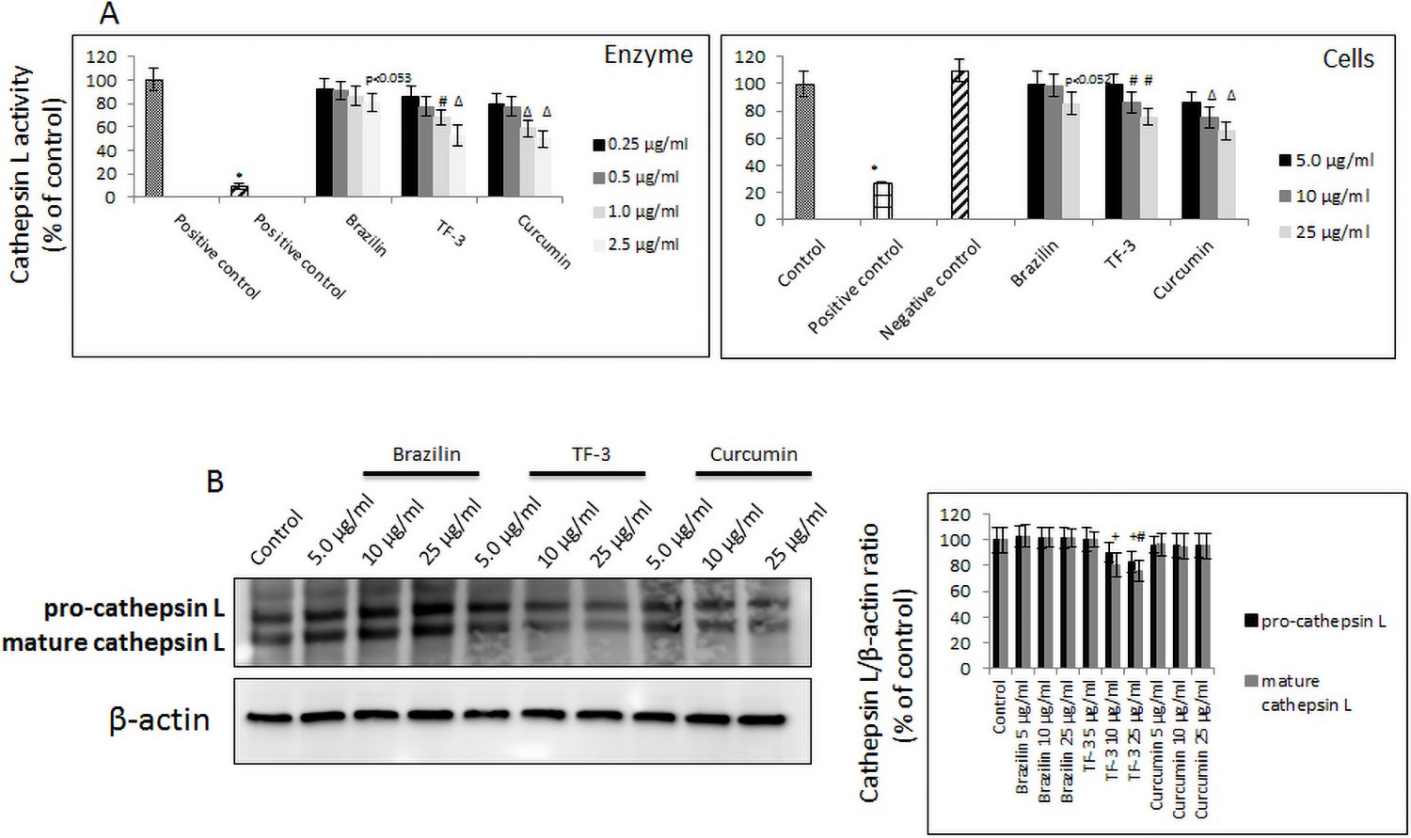
Effect of selected polyphenols on cathepsin L. (A) Activity of purified cathepsin L enzyme upon treatment with indicated polyphenols at different concentrations (left panel). Activity of cellular cathepsin L upon treatment with indicated polyphenols at different concentrations (right panel). Data are presented as % of control ± SD; Δ p ≤ 0.01, * p ≤ 0.001, + p < 0.054. Control– 0.025% DMSO, positive control– 0.1 μM E-64. (B) Western blot analysis of cathepsin L expression in A549 cells treated with indicated polyphenols with different concentration for 24h. (left panel) and quantified as band densitometry analysis indicating changes in protein expression (right panel). Data are presented as % of control ± SD; control– 0.025% DMSO, TF-3 –theaflavin-3,3’-digallate.

To further clarify if other components known to be involved in the SARS-CoV-2 virions’ cellular penetration, we checked activity and cellular expression of cathepsin L, utilizing human recombinant enzyme and enzyme derived from lysates of A549 cells treated with the tested polyphenols. In experiment with recombinant enzymes, curcumin proved to have the most profound inhibitory effect, ranging from 40% to 50% at 1.0–2.5 μg/ml. TF-3 followed, and showed 20% to 30% inhibition at 1.0–2.5 μg/ml, but brazilin had a minor, not significant effect. In cell lysates, we observed a similar trend, although inhibition of cathepsin L required 10 times higher concentrations of curcumin, which showed 20%-45% inhibition at 5.0–25 μg/ml, and TF-3, which revealed 20–25% inhibition at 10–25 μg/ml. Brazilin caused not significant 15% decrease at 25 μg/ml (Fig 6A). Interestingly, neither brazilin nor curcumin down-regulated cathepsin L expression at protein level, in contrast to TF-3, which modestly decreased its expression by about 20% starting from 10 μg/ml concentration (Fig 6B).

**Fig 7:**
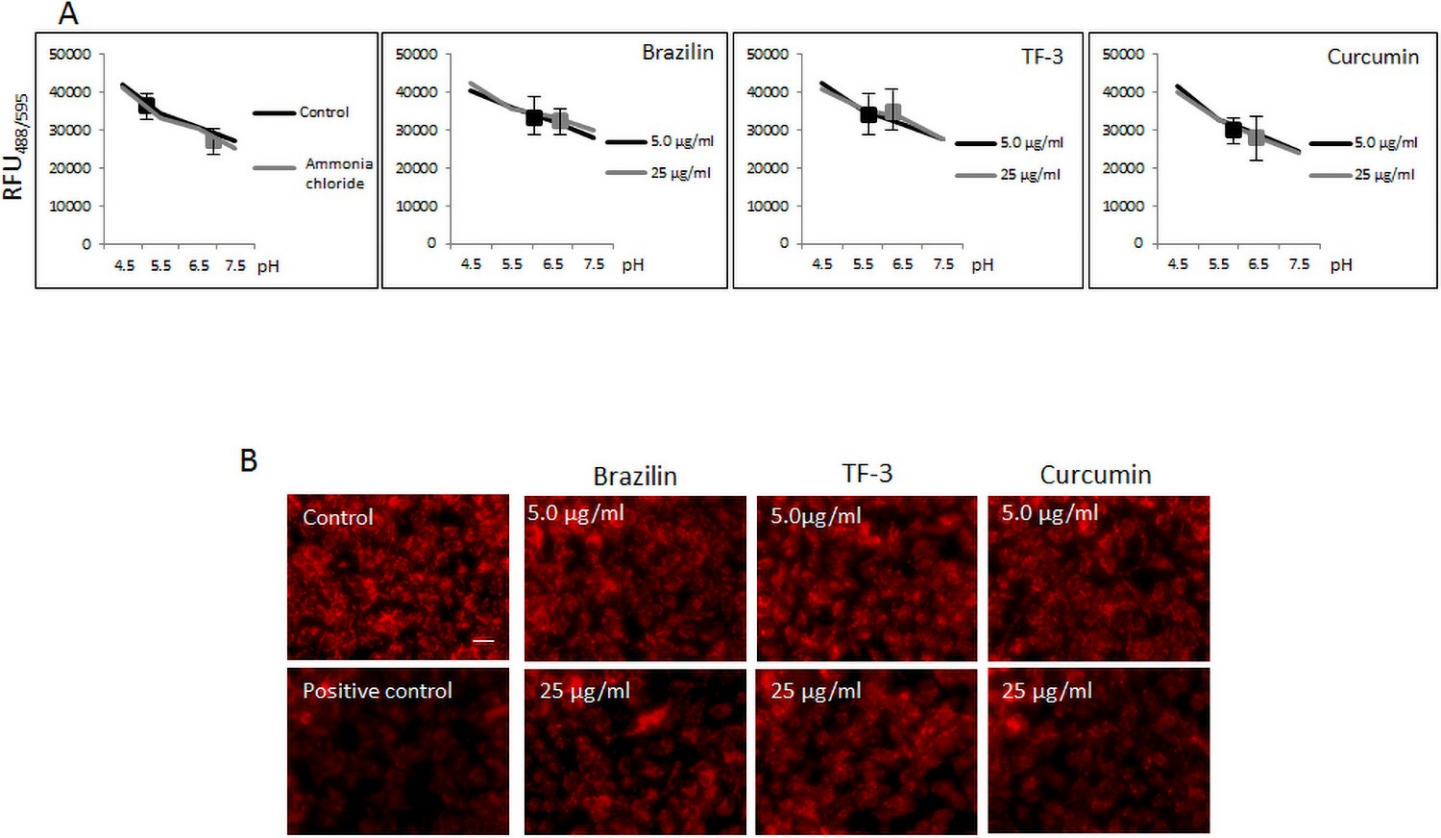
Effect of selected polyphenols on internal pH and endosome acidification. (A) Intracellular/lysosomal pH measurement. pHrodo^™^ Green AM dye and additional incubation for 30 min. at 37°C. Cells were then washed and fluorescence was measured at Ex/Em = 535/595 nm. Intracellular pH identification was done using standard curve prepared by measuring fluorescence in the presence of standard buffers with indicated pH as described in Material and Methods section. (B) Endosomal pH measurement in A549 cells treated with indicated polyphenols at different concentrations for 3h at 37°C. Scale bar indicates 50 μm. Images are representative of all observed fields. Experiments were done in triplicates and repeated three times. Data are presented as % of control ± SD. TF-3 –theaflavin-3,3’-digallate; control– 0.025% DMSO, positive control– 20 mM ammonia chloride.

Knowing that cathepsin L is a pH-sensitive protease, we employed 20 mM ammonia chloride as a positive control to check lysosomal/endosomal pH. Our results revealed that brazilin and curcumin can increase pH to about 6.0–6.5 at 5.0–25 μg/ml, whereas TF-3 elevates pH to about 5.5–6.0 at 5.0–25 μg/ml, compared with a control that, when measured, showed approximately pH = 5.0 (Fig 7A). This pattern was corroborated in the further experiment, where decreased fluorescence was observed upon treatment with these polyphenols at 5.0–25 μg/ml and acridine orange utilized as a pH sensor (Fig 7B).

## Discussion

Previous studies based on computational modeling and virtual screenings suggest that polyphenols mediate their anti-SARS-CoV-2 activity through diverse mechanisms [33]. For example, Wu *et al*. showed that theaflavin 3,3’-di-*O*-gallate, 14-deoxy-11,12-didehydroandrographolide, betulonal, and gnidicin exhibit high binding affinity to viral RdRp polymerase, whereas licoflavonol, cosmosiin, neohesperidin, and piceatannol target the binding between RBD of spike protein and hACE2, although it was predicted that only hesperidin would directly bind to the RBD of SARS-CoV-2 spike protein [34].

A study by Rehman *et al*. revealed that kaempferol, quercetin, and rutin were able to bind at the SBP (Substrate Binding Pocket) of 3CLpro with high affinity (i.e., 10^5^−10^6^ M^-1^), interacting with active site residues of 3CLpro such as His^41^ and Cys^145^ [35]. They also stated that the binding affinity of rutin was 1,000 times higher than that of chloroquine and 100 times higher than hydroxychloroquine. Based on the molecular docking study by Chen and Du, baicalin, scutellarin, hesperetin, nicotianamine, and glycyrrhizin have been identified as potential ACE2 inhibitors that could be used as possible anti-SARS-CoV-2 agents preventing its entry [36]. Compounds such as baicalin, (–)-epigallocatechin gallate, sugetriol-3,9-diacetate, and platycodin D revealed high binding affinity to the PLpro (papain-like protease) molecule that generates Nsp1, Nsp2 and Nsp3 proteins involved in the viral replication process [34].

According to Patel *et al*., curcumin and its derivatives showed high binding affinity to the RBD of SARS-CoV-2, with ΔG (i.e., binding energy) between -10.01 to -5.33 kcal/mol. Based on a binding energy that resembles that of synthetic drugs, and also pharmacokinetic parameters, these researchers identified curcumin as a candidate for SARS-CoV-2 spike protein inhibition [37]. Moreover, Jena *et al*. reported on catechin and curcumin, which have dual binding affinity, i.e., they bind to viral spike protein as well as to hACE2, although catechin’s binding affinity is greater (i.e., cathechin: -7.9 kcal/mol and -7.8 kcal/mol; curcumin: -10.5 kcal/mol and -8.9 kcal/mol, respectively) [38]. While these theoretical and molecular modelling approaches could identify potential applications of various molecules, the experimental proofs of their efficacy remain sparse.

Here, we provide *in vitro* evidence that among 56 tested phenolic compounds and plant extracts, brazilin, TF-3, and curcumin exhibited the highest binding to RBD-spike protein of SARS-CoV-2. Utilizing spike protein expressing hA549 cells we corroborated this result. By employing spike protein-enveloped pseudo-virions and different pattern of exposure, we observed in our short-term (i.e., exposure time 1 hour or 3 hours) and long-term studies (i.e., exposure time 48 hours), that all three compounds can inhibit viral attachment to the cell surface regardless of the time of exposure or incubation pattern. When the enveloped SARS-CoV-2 virions were pre-incubated with these compounds for 1h, added simultaneously, or when the compounds were added 1h post-infection to the cellular monolayer, their ability to bind to the ACE2 receptor and transduce was dose-dependently decreasing.

Interestingly, the same effect, although at higher but still non-toxic concentrations, was seen when SARS-CoV-2 pseudo-virions where forcibly attached to the cell surface by spin-inoculation. Additionally, we noticed that brazilin, TF-3, and curcumin can reduce fusion of spike-expressing cells to the hACE2 overdressing cellular monolayer. This confirmed our previous results indicating that all these three compounds have inhibitory properties directed especially towards RBD-SARS-CoV-2, and also suggest that they may also have an inhibitory effect on cellular proteases involved in SARS-CoV-2 infection steps. Our study did not elaborate on whether these polyphenols destroy viral particles, or whether they act by altering either the membranes of SARS-CoV-2 spike-enveloped pseudo-virions or the A549 cells. However, it has previously been shown that curcumin alters binding and fusion of the hepatitis C virus to the cell surface by affecting membrane fluidity [39].

Meanwhile, a study by Chen *et al*. documented that curcumin inhibits infectivity only of enveloped viruses, including the influenza virus [40]. Since curcumin is a lipophilic molecule, it can induce the morphological changes in a membrane, reflected in disturbed integrity and increased fluidity, which may alter the conformation of both viral and host proteins [41].

It has further been shown that theaflavins act as inhibitors of viral entry. For example, Chowdhury *et al*. found that theaflavins, including TF-3, inhibit the early steps of cellular entry of the hepatitis C virus, and suggested that they act directly on the viral particles rather than host cells blocking their dissemination [42]. Cui *et al*. specifically reported on theaflavin-3,3’-digallate as an inhibitor of serine protease NS2B-NS3 of the Zika virus [43]. Moreover, it was found from *in silica* study that theaflavins have a high binding affinity (i,e., ΔG of −8.53 kcal/mol) to the RBD of SARS-CoV-2 through forming hydrophobic interactions along with hydrogen bonds at ARG454, PHE456, ASN460, CYS480, GLN493, ASN501, and VAL503 of RBD-SARS-CoV-2, in proximity of the ACE2-spike protein contact area [44]. Also, Maiti and Banerjee reported that theaflavin gallate prevents the RBD spike protein from binding to the hACE2 receptor [45].

Based on our results that corroborate the other published data, we cannot exclude that polyphenols tested in our study may also induce, directly or indirectly, allosteric interaction affecting other molecules and processes involved in SARS-CoV-2 infectivity. Thus, our further experiments were focused on molecules facilitating binding and entry of SARS-CoV-2, such as ACE2, TMPRSS2, and cathepsin L [12, 21, 23]. Experiments in which the main attention was paid to ACE2 revealed that TF-3, and to a greater extent curcumin (but not brazilin), inhibit activity of ACE2 at non-toxic concentrations in both cell-free and cell-based assays. TF-3 and curcumin were shown to moderately bind to the hACE2 receptor at considerably low concentrations. Interestingly, none of these polyphenols down-regulated the expression of hACE2 at the protein level in A549 cells. This part of our study supports previously published computational prediction by Patel *et al*. and Jena *et al*. [37, 38]. Also, Zhang *et al*., through docking screening, found that TF-3 could directly bind to the ACE2 receptor [46].

With regards to TMPRSS2, our experimental results showed that brazilin, TF-3, and curcumin can decrease activity of TMPRSS2 in cell-free and cell-based assays, but precisely how they inhibit its enzymatic activity, which reflects in interference with virus binding to the cell surface, remains to be established. Interestingly, as with hACE2, the protein expression level of TMPRSS2 was not affected. Our results further showed that Tf-3 and, again more profoundly, curcumin inhibit activity of cathepsin L in cell-free and cell-based assays. To add to this, all of the selected polyphenols, albeit to different extents, increased lysosomal/endosomal pH from around pH = 5.0, concurring with previous reports [47], to around pH = 6.0–6.5. This could have the effect on activity of cathepsin L. However, with regard to TF-3 and especially curcumin, in either direct or close proximity, binding could happen, since upon treatment with TF-3 and curcumin, inhibition of cathepsin L activity was statistically significant, though only mildly down-regulated upon treatment with brazilin. The precise mechanism for this inhibition, reflected in the interference with viral endosomal egress, could be further clarified by utilizing computational study.

Ravish *et al*. recognized curcumin as an inhibitor of cathepsin B and H, and found a correlation with results obtained from the computational docking experiment [48]. In contrast to ACE2 and TMPRSS2 molecules, we observed also that TF-3, but not brazilin or curcumin, modestly decreased expression of cathepsin L at the protein level. Zhang *et al*. reported that curcumin increases the expression of cathepsin K and L in bleomycin-treated mice and human fibroblasts [49], while a study by Yoo *et al*. showed that expression of cathepsin L, elevated by palmitate in adipose tissue, can be inhibited by curcumin [50]. This suggests that it is a tissue- and cell-specific process.

Altogether, our results show that brazilin, TF-3, and curcumin can affect critical mechanisms involved in SARS-CoV-2 cellular entry and internalization. This study also expands our knowledge of the number of viruses that are sensitive to curcumin and TF-3, and identifies novel polyphenol brazilin with anti-SARS-CoV-2 properties, highlighting the mechanism by which these polyphenols can act to inhibit SARS-CoV-2 infectivity. It remains to be investigated whether other cellular and viral molecules that contribute to SARS-CoV-2 infection could be affected by these polyphenols. Application of this class of compounds might unravel previously unidentified but important mechanisms to expand our understanding of SARS-CoV-2 biology. Particularly interesting would be details behind their efficacy in SARS-CoV-2 pathophysiology during later steps of the infection process. It also raises a question as to whether these polyphenols could be detrimental or beneficial for host responses following SARS-CoV-2 infection, and whether their antiviral potential could support or complement current pharmacological treatment. Also, since the SARS-CoV-2 pseudo-virus and A549 cells that naturally express low levels of ACE2 were used in this study, further validation by utilizing primary alveolar type II epithelial cells and SARS-CoV-2 virus is warranted.

## Supporting information

S1 File Raw Western blot images. (PPTX)
