# Supplementary material for: Phenolic compounds disrupt spike-mediated receptor-binding and entry of SARS-CoV-2 pseudo-virions
Source: PLoS One. 2021 Jun 17;16(6):e0253489. doi: 10.1371/journal.pone.0253489 (PMC8211150; doi:10.1371/journal.pone.0253489)
Supplement: S1 File — (PPTX) [file pone.0253489.s001.pptx]

## Slide 1
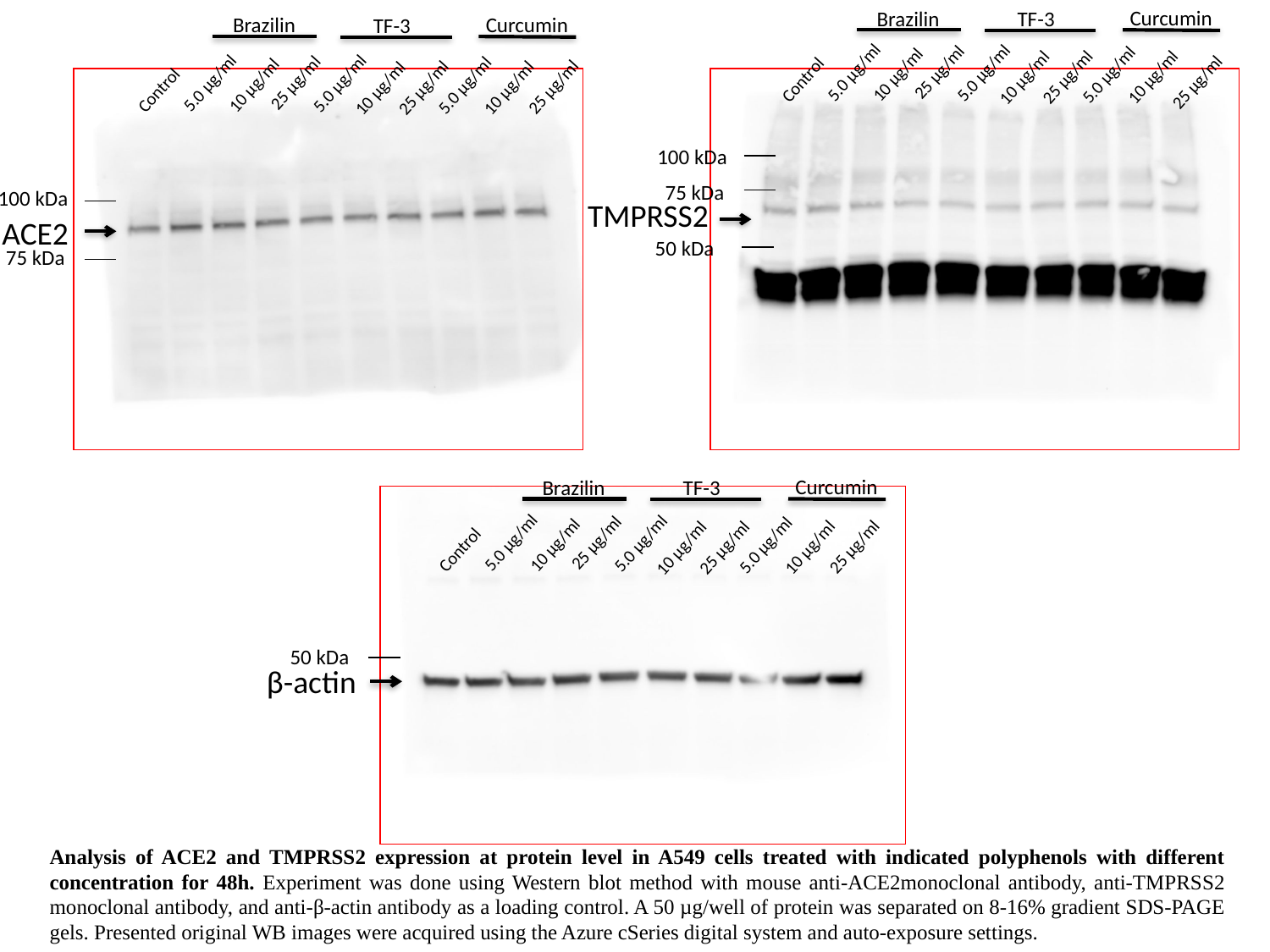

Curcumin
 Brazilin
 TF-3
Curcumin
 Brazilin
 TF-3
25 µg/ml
5.0 µg/ml
5.0 µg/ml
10 µg/ml
5.0 µg/ml
25 µg/ml
10 µg/ml
10 µg/ml
25 µg/ml
25 µg/ml
5.0 µg/ml
5.0 µg/ml
10 µg/ml
5.0 µg/ml
25 µg/ml
25 µg/ml
10 µg/ml
10 µg/ml
Control
Control
100 kDa
75 kDa
100 kDa
TMPRSS2
ACE2
50 kDa
75 kDa
Curcumin
 Brazilin
 TF-3
25 µg/ml
5.0 µg/ml
5.0 µg/ml
10 µg/ml
5.0 µg/ml
25 µg/ml
25 µg/ml
10 µg/ml
10 µg/ml
Control
50 kDa
β-actin
Analysis of ACE2 and TMPRSS2 expression at protein level in A549 cells treated with indicated polyphenols with different concentration for 48h. Experiment was done using Western blot method with mouse anti-ACE2monoclonal antibody, anti-TMPRSS2 monoclonal antibody, and anti-β-actin antibody as a loading control. A 50 µg/well of protein was separated on 8-16% gradient SDS-PAGE gels. Presented original WB images were acquired using the Azure cSeries digital system and auto-exposure settings.

## Slide 2
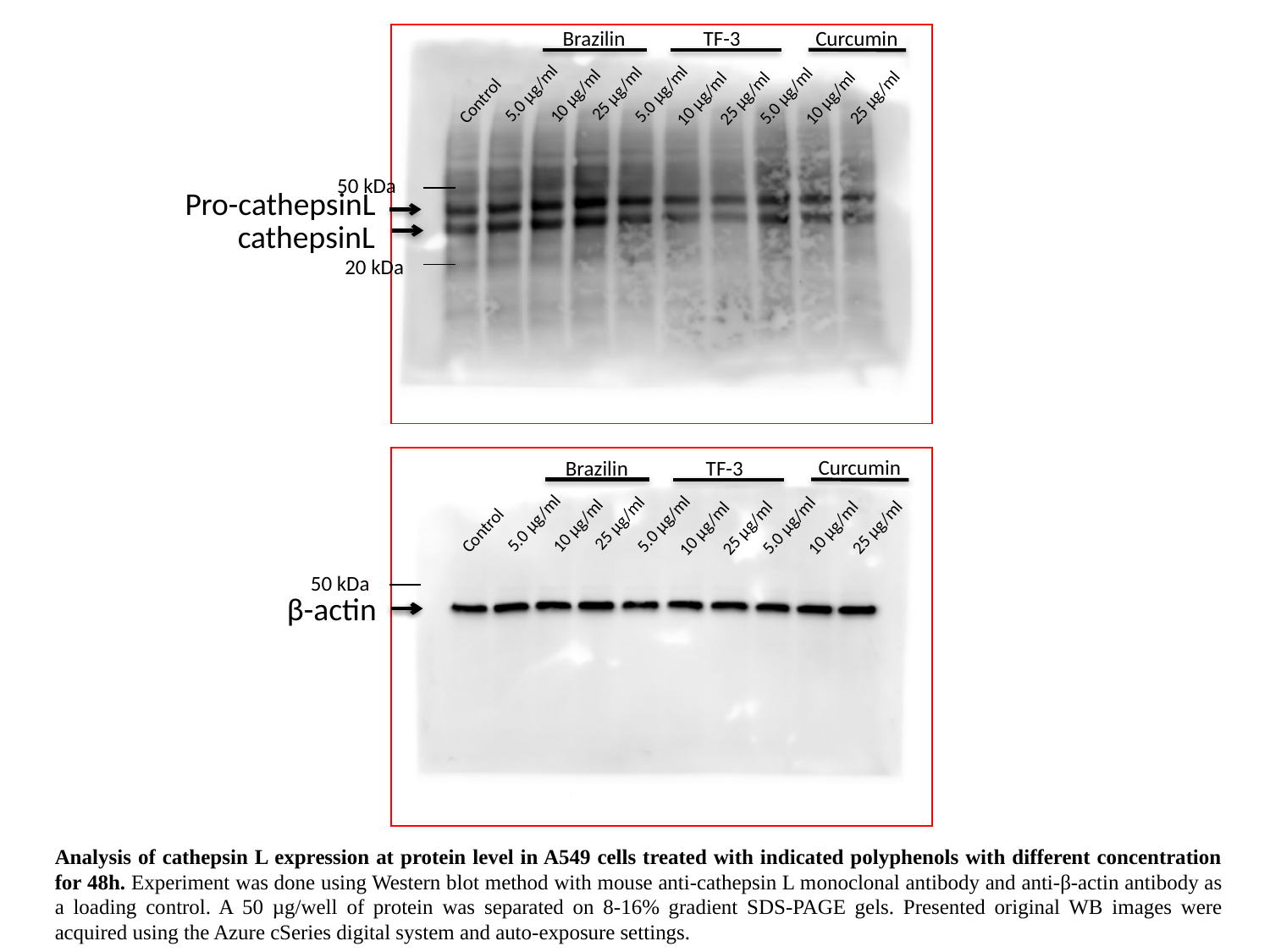

Curcumin
 Brazilin
 TF-3
25 µg/ml
5.0 µg/ml
5.0 µg/ml
10 µg/ml
5.0 µg/ml
25 µg/ml
25 µg/ml
10 µg/ml
10 µg/ml
Control
50 kDa
Pro-cathepsinL
 cathepsinL
20 kDa
Curcumin
 Brazilin
 TF-3
25 µg/ml
5.0 µg/ml
5.0 µg/ml
10 µg/ml
5.0 µg/ml
25 µg/ml
25 µg/ml
10 µg/ml
10 µg/ml
Control
50 kDa
β-actin
Analysis of cathepsin L expression at protein level in A549 cells treated with indicated polyphenols with different concentration for 48h. Experiment was done using Western blot method with mouse anti-cathepsin L monoclonal antibody and anti-β-actin antibody as a loading control. A 50 µg/well of protein was separated on 8-16% gradient SDS-PAGE gels. Presented original WB images were acquired using the Azure cSeries digital system and auto-exposure settings.
